# Unmasking the anti-gender mindset: development and validation of the Gender Ideology Belief Scale

**DOI:** 10.3389/fpsyg.2026.1931745

**Published:** 2026-09-16

**Authors:** Ivan Giuseppe Cammarata, Stefano Boca, Rocco Carmine Servidio, Martina Basilico

**Affiliations:** 1 Department of Psychology, Educational Science and Human Movement, University of Palermo, Palermo, Italy; 2 Department of Culture, Education and Society, University of Calabria, Arcavacata di Rende, Italy

**Keywords:** anti-gender discourse, gender ideology, LGBTQIA+, scale development, sexual prejudice

## Abstract

**Introduction:**

Opposition to gender ideology has become a socially and politically salient phenomenon, yet there is still a lack of validated instruments capable of capturing its multidimensional structure in a systematic way. The present research aimed to develop the Gender Ideology Belief Scale (GIBS), a new measure designed to assess negative attitudes toward gender ideology as a coherent ideological construct.

**Methods:**

Across two studies involving Italian participants (total *N* = 595), the GIBS was developed through a multi-phase process including literature review, analysis of public anti-gender discourse, item generation, expert evaluation, and psychometric testing. In Study 1 (*N* = 332), exploratory factor analysis was used to identify the most suitable items and examine the latent structure of the scale, whereas in Study 2 (*N* = 263), confirmatory factor analysis was conducted to test the factorial structure and assess reliability, construct validity, and criterion validity.

**Results:**

The exploratory factor analysis supported a three-factor solution reflecting conspiratorial beliefs, resistance to gender-inclusive education, and adherence to traditional family values. Confirmatory factor analysis supported a second-order structure in which these three dimensions loaded onto a broader gender ideology factor, and the final 14-item version of the scale demonstrated acceptable reliability and associations with right-wing authoritarianism, sexual prejudice, and conspiracist beliefs.

**Discussion:**

The GIBS provides researchers with a new instrument for examining opposition to gender ideology as an organized belief system rather than as an isolated attitude. By offering a multidimensional measure of anti-gender beliefs, this scale may support future research on cultural backlash, ideological resistance to social change, and the psychological dynamics underlying contemporary conflicts over gender and diversity.

## Introduction

Gender ideology has emerged as a central topic in contemporary academic and public discourse, reflecting broader debates about identity, equality, and social norms. Rooted in feminist theory and gender studies, the term initially gained traction through the work of scholars such as Simone de Beauvoir (1949), who famously asserted in *The Second Sex* that “*one is not born, but rather becomes, a woman*” (p. 267), emphasizing the social construction of gender and challenging the notion that biology alone determines one’s destiny. This insight laid the groundwork for subsequent scholarship that sought to disentangle gender from biological sex, highlighting the role of cultural, historical, and social factors in shaping gender identities and roles (Oakley, 1972; West and Zimmerman, 1987). The 1990s marked a significant turning point with the publication of Judith Butler’s *Gender Trouble* (1990), which introduced the concept of gender as a performative act: a series of repeated behaviors and expressions that create the illusion of a stable gender identity (p. 25). Butler’s work challenged the binary understanding of gender as strictly tied to biological sex, opening up new avenues for exploring non-binary and fluid gender identities. Over the past few decades, these theoretical developments have profoundly influenced public policies and social practices aimed at promoting gender equality and LGBTQIA+ rights.

However, the term underwent a semantic transformation that is essential to understanding what is measured here: it did not originate within academic gender studies but was constructed as a political device. Its earliest traceable roots lie in the activism of O'Leary (1997), who reframed gender, in the wake of the 1995 Beijing conference, as a coded synonym for a progressive agenda attributed to a coalition of elites (see also Sommers, 1994); the framing was then institutionalized through Catholic Church documents and subsequently migrated into secular right-wing politics (Garbagnoli, 2016; Garbagnoli and Prearo, 2017; Graff and Korolczuk, 2022). As Butler (2024) observes, the expression functions as a political phantasm: an emotionally charged and semantically indeterminate signifier that condenses heterogeneous anxieties about family, national identity, and moral order into a single, conspiratorial threat.

These claims have had tangible effects on policy and education. Mobilizations against gender-related educational initiatives have occurred in Italy in 2013 and again in 2021 around the Ddl Zan (Feo, 2022; Garbagnoli, 2016; Lavizzari and Prearo, 2019), in France around La Manif Pour Tous (Stambolis-Ruhstorfer and Tricou, 2017; Fassin, 2016), and in Poland, where gender studies have been labeled an ideology and defunded (Graff and Korolczuk, 2022), in several cases leading to the withdrawal or modification of educational materials (Corredor, 2019; Kuhar and Paternotte, 2017). What these cases share is not simply a policy stance but a recognizable set of claims about what gender ideology is, who is held to promote it, and what it is said to threaten: claims that are psychologically organized and, we argue, measurable. It is this psychological organization, rather than the political history of the term, that the present research addresses.

### Belief systems and attitudes toward gender ideology

Belief systems, understood as organized frameworks of knowledge, values, and assumptions that shape how individuals interpret the world, are central to understanding societal responses to contested issues like gender ideology. Rooted in social psychology, belief systems function as cognitive filters, guiding perceptions, attitudes, and behaviors. They are often reinforced by cultural, religious, and institutional narratives, creating a common sense that endorses certain worldviews while marginalizing others (Fishbein and Ajzen, 2010). Within this framework, attitudes toward social groups, including sexual minorities, are not independent judgments but rather downstream expressions of broader ideological commitments embedded in one’s belief system (Herek, 1987; Jost et al., 2004, 2009).

The debate over gender ideology illustrates how belief systems do not merely reflect pre-existing values, but actively construct the meaning attributed to social phenomena. For individuals embedded in worldviews that privilege tradition, hierarchy, and biological essentialism, gender ideology is not simply a theoretical framework to be evaluated, but an intrusion into a moral order perceived as natural and divinely sanctioned (Garbagnoli, 2016; Corredor, 2019). Conversely, those operating within egalitarian belief systems interpret the same discourse as a necessary corrective to structural inequalities, drawing on a tradition that situates gender as a historically produced and transformable category (Butler, 1990; Fishbein and Ajzen, 2010). It is within the former type of belief system, organized around tradition, authority, and moral purity, that opposition to gender ideology finds its most coherent psychological grounding. These groups frequently portray gender ideology as a destabilizing force, invoking concerns about purity, natural order, and moral contamination, themes that resonate with the Purity/Sanctity foundation identified within Moral Foundations Theory (Haidt and Graham, 2007; Graham et al., 2013). According to this framework, individuals who place greater weight on foundations of Authority and Purity tend to evaluate non-normative gender identities and sexual orientations as threats to the natural and moral order, thus producing more negative evaluative responses toward LGBTQIA+ individuals (Haidt, 2012).

What is commonly labeled as homophobia or sexual prejudice (Herek, 2004) can thus be understood not as a discrete psychological trait, but as a predictable output of specific belief systems that frame heterosexuality and binary gender as natural, divinely sanctioned, and socially necessary. From this perspective, negative attitudes toward gay men and lesbians emerge as coherent expressions of a broader ideological worldview rather than as isolated or irrational reactions (Herek, 1987; Jost et al., 2004). Indeed, research grounded in System Justification Theory has shown that homonegativity functions as a legitimizing myth of gender hierarchy, reinforcing the perceived fairness and naturalness of heteronormative arrangements (Ferrari et al., 2021). Similarly, right-wing authoritarianism, which reflects deference to authority and adherence to social conventions, consistently predicts sexual prejudice precisely because it reflects a belief system organized around the preservation of established hierarchies and moral norms (Altemeyer, 1996; Whitley, 1999; Hoffarth et al., 2023). In this sense, the opposition to gender ideology and the expression of homonegativity represent two intertwined manifestations of the same underlying system of beliefs about social order, nature, and moral legitimacy.

Attitudes toward gender ideology are deeply embedded within competing belief systems, reflecting broader ideological battles over social norms and values. Defined as evaluative judgments ranging from favorable to unfavorable (Eagly and Chaiken, 1993), these attitudes are not merely personal opinions but may be shaped by divergent worldviews. On one side, individuals anchored in belief systems that prioritize tradition and hierarchy often perceive gender ideology as a potential moral threat, sometimes associating it with conspiracy theories (e.g., “global elites imposing a hidden agenda”) and expressing concerns about societal destabilization, particularly through education systems that promote gender diversity (Paternotte and Kuhar, 2018; Buss et al., 2020). This negative framing tends to revolve around three interconnected themes: (1) the notion of a covert agenda to undermine cultural norms, (2) concerns about children’s “indoctrination” via inclusive education, and (3) the perceived erosion of traditional family structures (Garbagnoli, 2016; Graff and Korolczuk, 2022). For example, opponents frequently claim that gender ideology is being advanced by international organizations or political elites, framing initiatives like LGBTQ+ inclusive curricula as potential threats to “natural” gender roles and familial stability (Verloo, 2018). Conversely, individuals with an egalitarian ideological orientation may perceive gender ideology as a pivotal framework for challenging oppressive structures and promoting equality.

Framing opposition to gender ideology as the expression of a belief system has direct consequences for the way it should be measured. If the three domains outlined above are surface manifestations of a shared ideological orientation, then the appropriate operationalization is a reflective measurement model, in which the covariation among items is generated by the latent constructs they indicate rather than being defined by them (Bollen and Lennox, 1991). Because the three domains are themselves expected to covary as facets of a superordinate construct, a hierarchical specification, with domain-specific first-order factors accounted for by a general second-order factor, provides the most direct translation of the theoretical model into a testable measurement structure (Chen et al., 2006; Johnson et al., 2011). This correspondence between construct definition and model specification is a core requirement of contemporary validation practice (Boateng et al., 2018; Clark and Watson, 2019; Flake et al., 2017): a substantive claim about how a belief system is organized should be evaluated rather than assumed, by comparing the hypothesized structure with theoretically plausible alternatives. We report such a comparison in Study 2.

### Developing a measure of gender ideology

There is a lack of reliable and valid instruments to measure the attitudes associated with the negative perception of gender ideology. Existing scales do not fully capture the unique dimensions of this phenomenon, particularly the core claims that gender ideology seeks to collapse natural differences, posing a threat to children and the traditional family and that it represents an imposition from global elites.

This gap is substantive rather than merely instrumental, and it becomes apparent as soon as the existing quantitative evidence on gender backlash is examined closely. Much of what is known about the diffusion and the correlates of anti-gender attitudes comes from secondary analyses of large cross-national surveys such as the World Values Survey and the European Social Survey. This body of work has been highly generative: Norris and Inglehart (2019) drew on such data to formulate the cultural-backlash thesis, according to which authoritarian-populist mobilization is fuelled by the reaction of traditionalist constituencies against long-term value change; Peral et al. (2024) documented a stalling and, in several European countries, a partial reversal of the liberalization of gender-role attitudes, raising the question of whether gender has become a new axis of polarization; and Ural (2026) traced the way anti-gender discourse is converted into homonegative values in the Turkish context. Precisely because these contributions rely on pre-existing datasets, however, they must assess gender attitudes through a small number of legacy indicators, typically single items concerning the desirability of maternal employment, male priority in access to jobs, or the justifiability of homosexuality. Such indicators were designed to capture generalized traditionalism in gender roles, and they do so effectively, but they are unidimensional by construction and they predate the anti-gender mobilizations of the past two decades. As a consequence they cannot register what is distinctive about contemporary opposition to gender ideology: its conspiratorial framing of an externally orchestrated agenda, its concentration on children and schooling as the primary site of contention, and its self-presentation as the defence of a natural order rather than as prejudice toward a group. The GIBS is intended to address exactly this discrepancy between the phenomenon described in qualitative and political-science scholarship and the indicators currently available to quantitative research, by providing a multidimensional instrument whose content is derived from anti-gender discourse itself.

Given this gap, the present study proposes the development and validation of a multidimensional scale specifically designed to measure negative perceptions of gender ideology: the Gender Ideology Belief Scale (GIBS). Unlike previous instruments, the GIBS systematically integrates key elements of anti-gender discourse, ensuring a comprehensive assessment of opposition to gender-related social change.

The GIBS is structured around three core factors that encapsulate the dominant themes in anti-gender discourse, each reflecting broader socio-political concerns and resistance to shifts in gender norms.

The first factor, conspiracy, encompasses the perception that gender ideology is a deliberate strategy orchestrated by powerful elites or international organizations. This perspective frames gender discourse as a form of social engineering designed to erode cultural traditions, national sovereignty, and established moral values. Such narratives position gender-related policies and initiatives as external impositions that threaten the fabric of society, reinforcing a sense of collective victimization and distrust toward global institutions (Garbagnoli and Prearo, 2017).

The second factor, education, captures opposition to the integration of gender-related topics into educational curricula. This resistance is often rooted in the belief that such content could disrupt children’s cognitive and emotional development, undermine parental authority, and challenge religious or cultural values. Opponents argue that discussions on gender diversity and sexual orientation should remain within the purview of the family rather than be institutionalized within formal education (Schettini, 2023).

The third factor, values, reflects a strong commitment to conventional family structures and gender roles, which are perceived as being under attack by gender ideology. This perspective emphasizes the importance of binary gender distinctions, heterosexual marriage, and clearly defined roles for men and women as fundamental to social stability and moral order. The promotion of alternative family models or non-normative gender identities is frequently interpreted as a direct challenge to these traditional foundations, fueling resistance to policies or discourses advocating for greater gender inclusivity and diversity (Spallaccia, 2020).

Together, these three factors illustrate the multifaceted nature of anti-gender discourse, demonstrating how opposition to gender-related social change remains deeply intertwined with broader anxieties about cultural transformation, institutional authority, and the perceived erosion of traditional societal structures.

### Item generation

The GIBS items were generated through a four-phase procedure designed to maximize theoretical coherence, content relevance, and ecological validity; the full description of each phase is reported in Supplementary Material S1. In brief, we first reviewed the literature on anti-gender discourse across social and political psychology, gender studies, and adjacent fields, in order to identify its recurrent themes, symbolic oppositions, and rhetorical patterns. Second, we analyzed publicly available materials in which such discourse circulates in naturalistic settings, namely political speeches, media reports, movement manifestos, and statements issued by religious and conservative advocacy groups, so that item content would reflect the language actually used in public debate rather than abstract theorization alone. Both sources converged on the three domains described above. Third, we drafted an initial item pool covering the three domains and varying in tone and intensity, from explicitly conspiratorial claims to the more socially normalized expressions of concern about education, morality, and family through which anti-gender positions are frequently voiced. Fourth, the pool was submitted to expert evaluation by scholars in psychology, political science, and gender studies, who assessed the conceptual clarity, representativeness, and dimensional correspondence of each item; on the basis of their feedback several items were revised or discarded in order to improve precision and reduce redundancy. This process yielded the 24 items administered in Study 1.

## The present studies

The objective of this research is to create and validate a measure of a multidimensional scale specifically designed to measure negative perceptions of gender ideology: the Gender Ideology Belief Scale (GIBS). Consistent with an increasing number of calls for a more rigorous approach to the validation of psychological measures (Flake et al., 2017), we adhered to the recommended best practices for scale development (Boateng et al., 2018). We conducted two studies in which all participants were residents of Italy. The two studies adhered to the 1964 Helsinki Declaration and its subsequent amendments. We also wish to be explicit about the intended scope of the instrument. Both studies were conducted in Italy, and the GIBS is presented here as an Italian-language measure developed for, and validated in, the Italian sociopolitical context. Its use in other cultural settings is treated as an empirical question that requires adaptation and formal tests of measurement invariance rather than as an assumption, and we specify in the Limitations section the protocol we regard as necessary before such use.

In Study 1 (*N* = 332) we submitted the pool of items that we have created specifically to participants, and we analyzed their responses using an exploratory factor analysis to select the most suitable item.

In Study 2 (*N* = 263) we administered the newly created scale to participants to: (a) confirm its factorial structure using a confirmatory factor analysis; (b) assess its construct validity testing whether the assumed factorial structure fits the data, indicators such as CFI, TLI and RMSEA are used; (c) to assess its internal consistency Cronbach’s α and McDonald’s Ω are used; (d) to assess its criterion validity previous literature found that several socio-demographic (i.e., male gender, older people and low education), high religiosity, right-wing political orientation, and high levels of right-wing authoritarianism (RWA) predicted stronger opposition to progressive social policies, greater endorsement of traditional social norms and high levels of conspirative belief (Adelman et al., 2021; Douglas and Sutton, 2023; Duriez and Van Hiel, 2002; Godø et al., 2024; Salvati et al., 2024; Woodford et al., 2012; Worthen et al., 2017).

## Study 1

### Method

#### Participants and procedures

The primary objective of Study 1 was to conduct an exploratory factor analysis of the GIBS scale. As recommended by Howard (2016), the sample size was established with the aim of achieving an adequate participant-to-variable ratio of at least 5:1. Therefore, an initial pool of 24 items was administered to 347 participants. To check whether attention was maintained during completion, we included two control variables in the questionnaire. There were 15 subjects who did not give a correct answer and were therefore excluded from the sample. The analysis was performed on 332 subjects (M_Age_ = 31.48, SD_Age_ = 12.18; female = 56%, male = 43%, other = 1%; 40.2% have obtained a diploma or a lower qualification, 31.2% have attained a bachelor’s degree, 18.6% have obtained a master’s degree and 7.5% have obtained a doctorate or specialization). To recruit participants for the study, snowball sampling was employed. This method was chosen due to its effectiveness in accessing a diverse and relevant population for exploratory research. Initial participants were identified and subsequently asked to refer others who met the eligibility criteria: (a) Italian nationality and (b) at least 18 years old.

#### Measures

The questionnaire was administered using Google Forms, a secure online survey platform. Participants were provided with an information sheet at the beginning of the survey, which outlined the purpose of the study, the voluntary nature of their participation, and their right to withdraw at any time without penalty. Informed consent was obtained electronically before participants could proceed to the questionnaire.

The questionnaire consisted of two main sections: the first section included the Gender Ideology Belief Scale, while the second section collected socio-demographic information. The survey was designed to be concise, with an estimated completion time of approximately 6 min. Responses were anonymized to ensure participant’s confidentiality.

After answering the 24 items of the GIBS, the participants were invited to provide information regarding their gender, age, sexual orientation, educational level and political orientation. Participants were invited to indicate their gender by selecting one of three options: male, female or other (see Supplementary Table A1 for statistics). If the participant selected ‘other’, they were invited to provide a further explanation in the space provided. In a similar manner, subjects were invited to disclose their sexual orientation by selecting one of five alternatives (heterosexual, homosexual, bisexual, I do not answer and other). In the event of selecting ‘other’, subjects were invited to specify their sexual orientation. Finally, participants were invited to indicate their political orientation using a single item on a 9-point scale ranging from 1 = extreme left to 9 = extreme right.

Data collection was conducted entirely online, and the study received ethical approval from the University of Palermo (parere n. 294/2025, protocollo n. 40189-2025) prior to its commencement.

### Results

#### Exploratory factor analysis (EFA)

Analyses were performed using the RStudio graphical interface (R Core Team, 2021) and its psych (Revelle, 2025) and lavaan (Rosseel, 2012) packages. The normality of the distribution for the original pool of 24 items was tested before proceeding to examine the factorial structure of the GIBS. The limits of skewness and kurtosis were ±2.00 and ±7, respectively, (Curran et al., 1996). One item had values above these parameters, item ed5 (see Supplementary Table A2) and was therefore excluded from the EFA. Hence, the remaining 23 items did not show high skewness and kurtosis.

Preliminary testing was conducted in order to evaluate the suitability of the data for EFA. We first performed Bartlett’s test of sphericity (Bartlett, 1950). Results suggested a factorable matrix correlation, with the correlation matrix derived from the data differing significantly from a randomly generated correlation matrix, χ ^2^(253) = 5220.27, *p* < 0.001. Secondly, a Kaiser-Meyer-Olkin (KMO) test was performed (Kaiser, 1960). This provided evidence that the data was factorable, with very strong relationships among the variables (KMO = 0.94). The preliminary analysis thus indicated that the data was suitable for EFA.

The factor retention process was executed in accordance with the Kaiser criterion, the scree test (Cattell, 1966), and the parallel analysis (Glorfeld, 1995; Horn, 1965). The analyses revealed that four factors had an eigenvalue greater than 1, following Kaiser criterion. The first factor had an eigenvalue of 10.44, followed by the others: 2.26, 1.70 and 1.17. In the scree plot analysis, the eigenvalue curve flattened after the third factor (see Supplementary Figure A1). Horn’s Parallel Analysis suggests retaining three factors. This decision is based on the adjusted eigenvalues: component 1 (9.94), component 2 (1.84) and component 3 (1.34) are greater than 1, indicating they explain meaningful variance in the data. Components with adjusted eigenvalues below 1 are typically not retained (Glorfeld, 1995).

In order to investigate the factorial structure of GIBS, a principal axis factoring (PAF) extraction was implemented, with a three-factor solution selected based on the results of the parallel analysis and the scree test, in addition to the prevailing theoretical framework.

The 23-item pool was first examined in order to determine the loading of each item on the three factors. Items loading on the education factor showed loadings ranging from 0.93 to 0.72, items loading on the conspiracy factor from 0.89 to 0.42, and items loading on the values factor from 0.73 to 0.54 (see Supplementary Table A3). Given the recommendations for concise measures that can be administered without unduly burdening respondents, only the items with the strongest loadings, and therefore the most representative content for each factor, were retained for a second EFA. Item retention followed three criteria applied jointly: a primary loading of at least 0.40 on the intended factor, no cross-loading of 0.30 or above on any non-target factor, and a communality indicating a non-negligible proportion of explained variance. The 0.40 threshold was not chosen arbitrarily. It corresponds to approximately 16% of variance shared between item and factor, it comfortably exceeds the loading required for statistical significance at the present sample size (Stevens, 2009), and it is the value most consistently recommended in methodological reviews of exploratory factor analysis and in scale-development practice (Comrey and Lee, 1992; Hair et al., 2019; Howard, 2016). Applying a more permissive threshold would have retained items with less than 10% of variance in common with their factor, whereas a more restrictive one would have reduced the content coverage of the conspiracy dimension.

Adhering to this established criterion, the selection process was undertaken, resulting in the identification of a total of 15 items. Subsequently, an extra EFA was conducted to evaluate the finalized three-dimensional configuration of GIBS. The analysis indicated three factors with a value greater than 1, accounting for 70% of the variance. This finding was corroborated by the visual scree test and parallel analysis (see Supplementary Figure A2). The factor loadings of the items ranged from 0.88 to 0.56 (see Table 1).

**Table 1:** Factor loadings (λ) and communalities (h^2^) of the 15 items retained in the exploratory factor analysis (Study 1).

Item	Item content	Com	Val	Ed	h2
Com3	Rich people decide the rules about what is masculine and what is feminine.	0.64			0.31
Com5	TV and newspapers want to change people’s minds about what it means to be male or female.	0.86			0.77
Com6	International organizations are trying to create confusion about what it means to be male or female.	0.66			0.40
Com7	Changes in the roles of men and women are being imposed by those in power.	0.69			0.60
Com8	There is a global movement aiming to eliminate the natural distinctions between men and women.	0.75			0.82
Val1	The family unit functions best when men and women take on distinct roles		0.74		0.69
Val2R	Believing that gender identities other than male and female exist can be compatible with religious traditions.		0.56		0.23
Val5	I consider marriage between a man and a woman to be an important value for our society.		0.72		0.69
Val6	For me, the distinction between male and female is a natural and unchanging reality.		0.88		0.82
Val7	I believe that cultural and moral traditions relating to the family should be respected and passed on.		0.76		0.67
Ed1R	Addressing gender diversity at school contributes to the development of empathy in students			0.81	0.72
Ed3	Discussing gender-related issues at school risks causing confusion among children.			0.88	0.69
Ed4	I believe that gender education in schools should reflect traditional family values			0.88	0.65
Ed6R	Teaching new perspectives on male and female roles enriches education.			0.78	0.69
Ed8R	Schools should update their content on gender to reflect current scientific knowledge.			0.58	0.40

*N* = 332. Principal axis factoring; loadings below 0.20 are suppressed. Com, conspiracy; Val, values; Ed, education; h^2^, communality. Items ending in R are reverse-scored. Item Val2R was subsequently removed on the basis of the Study 2 confirmatory analysis (see text).

Finally, an investigation was conducted into the reliability of the 15 items and three-dimensional solution of GIBS. In view of the recent criticism leveled at the alpha coefficient (Deng and Chan, 2017), an assessment of reliability was undertaken using both Cronbach’s α and McDonald’s Ω. The analyses revealed excellent values, specifically for the conspiracy factor α = 0.85, Ω = 0.89, the values factor α = 0.88, Ω = 0.90 and the education factor α = 0.89, Ω = 0.91.

## Study 2

### Method

#### Participants and procedures

In Study 2 (*N* = 263), the newly developed scale was administered to assess its factorial structure (via CFA), construct validity (CFI, TLI and RMSEA), and internal and criterion validity (Cronbach’s α, McDonald’s Ω and associations with socio-demographic and ideological variables linked to resistance to progressive social change). An *a priori* power analysis tailored to structural equation modeling was conducted using the semPower package (Moshagen and Bader, 2023) to determine the required sample size for the confirmatory factor analysis, based on the approach proposed by Moshagen and Erdfelder (2016). Setting RMSEA at 0.05, α at 0.05 and power at 0.80 a second-order factor model composed of 15 observed variables loading onto three first-order latent factors, each in turn loading onto a single second-order latent factor was tested. The analysis indicated that a minimum sample size of 152 participants was required to achieve sufficient statistical power (see Supplementary Figure A3).

Participants in Study 2 were aged 30.61 on average (SD = 13.48), and identified as female 69.2%, male 28.1% or other 2.7%. Participants’ educational backgrounds varied, with 57.8% having obtained a diploma or lower qualification, 12.9% holding a bachelor’s degree, 26.6% a master’s degree and 2.7% a doctorate or specialized qualification. Recruitment was carried out using a snowball sampling technique, selected for its ability to reach a broad and relevant population. The initial cohort of participants was recruited and subsequently asked to refer other individuals who satisfied the inclusion criteria of being of Italian nationality and being at least 18 years old.

#### Measures

The questionnaire comprised four scales, which were presented in a randomized sequence after administering the 15-item version of the GIBS scale. Sociodemographic variables, collected at the end of the questionnaire, were consistent with those employed in Study 1 (see Supplementary Table A4).

The following measures were included:

Right Wing Authoritarianism (RWA): RWA was measured using 14 items of the Italian RWA scale by Giampaglia and Roccato (2002) adapted from Altemeyer (1996; for example: “Our country needs a powerful leader to destroy the radical and immoral currents prevailing in society today”). Items were answered on five-point scales ranging from −2 (strongly disagree) to +2 (strongly agree). Cronbach’s α was 0.89.

Modern homonegativity against homosexuals: We used the Modern Homonegativity Scale - Gay men (MHS-G, Morrison, 2003). Twelve items measured the discrimination against homosexuals in modern society (for example: “Gay men who are “out of the closet” should be admired for their courage,” and “Gay men have become far too confrontational in their demand for equal rights”). Items were answered on five-point scales ranging from 1 (strongly disagree) to 5 (strongly agree). Cronbach’s α was 0.93.

Old-fashioned homonegativity against homosexuals: To measure hostility against homosexual people we used the Attitude Towards Gay Men Scale (ATG-S; Herek, 1994). Five items measured old-fashioned homonegativity (for example, “Male homosexuality is merely a different kind of sexual orientation that should not be condemned”, and “I think male homosexuals are disgusting”). Items were answered on five-point scales ranging from 1 (strongly disagree) to 5 (strongly agree). Cronbach’s α was 0.85.

Generic Conspiracist Beliefs Scale (GCBS): GCBS was measured using the 14-item Italian version of the Generic Conspiracist Beliefs Scale, adapted from Brotherton et al. (2013); the Italian version was validated by Antichi et al. (2023). The scale assesses the tendency to engage in conspiracy theories (conspiracist ideation). An example item is: “A small, secret group of people is responsible for making all major world decisions, such as going to war”. Items were answered on a 5-point Likert scale ranging from 1 (strongly disagree) to 5 (strongly agree). Cronbach’s α was 0.92.

### Results

#### Confirmatory factor analysis (CFA)

The confirmatory factor analysis (CFA) was performed using the lavaan package in R (Rosseel, 2012; R Core Team, 2021). Prior to conducting the CFA, the distributional properties of the fifteen items comprising the GIBS were examined. Descriptive statistics and normality indices were computed for all items prior to the CFA. Means ranged from 1.57 to 3.00, while standard deviations varied between 1.01 and 1.44, indicating sufficient variability across items. Like for Study 1, distributions were considered sufficiently normal when skewness did not exceed ±2.00 and kurtosis remained within ±7.00 (Curran et al., 1996). All items met these criteria, with skewness values ranging from 0.00 to 1.95 and kurtosis values ranging from −1.20 to 3.23, suggesting no substantial departures from normality (see Supplementary Table A5). On this basis, the data were deemed appropriate for analysis using the robust maximum likelihood estimator (MLR) in the subsequent CFA.

A second-order confirmatory factor analysis (CFA) was conducted to test the hypothesized hierarchical structure in which three first-order latent constructs: (1) conspiracy factor, (2) education factor, and (3) values factor, were accounted for by a higher-order latent factor labeled gender ideology. The model showed an acceptable fit to the data, χ^2^ (87) = 193.78, *p* < 0.001, robust CFI = 0.894, robust TLI = 0.872 and robust RMSEA = 0.074 (90% CI [0.060, 0.088]), and SRMR = 0.067. All factor loadings were statistically significant (*p* < 0.001). The higher-order factor was strongly related to both education factor (standardized λ = 0.87) and values factor (λ = 0.92) and moderately associated with conspiracy factor (λ = 0.53). While the model’s global fit indices were slightly below conventional thresholds for excellent fit, the overall structure was empirically supported. One item from the values factor (Val2R) showed a particularly low standardized loading (0.25), suggesting possible item-level limitations.

Given the particularly low standardized loading of item Val2R, a second confirmatory factor analysis was conducted after removing this item from the model. The revised second-order model, excluding Val2R, demonstrated a comparable and slightly improved fit, χ^2^ (74) = 169.21, *p* < 0.001, robust CFI = 0.903, robust TLI = 0.880, and robust RMSEA = 0.076 (90% CI [0.061, 0.091]), and SRMR = 0.064.

All remaining items continued to load significantly on their respective latent factors (*p* < 0.001), and the higher-order structure was preserved, with strong standardized loadings from values factor (λ = 0.93) and education factor (λ = 0.86), and a moderate loading from conspiracy factor (λ = 0.54) (see Table 2 and Figure 1).

**Table 2:** Standardized factor loadings of the confirmatory factor analysis of the 14-item version of the GIBS (Study 2).

Item/factor	Item content	*β*	*SE*	*z*	*p*
Conspiracy					
Com3	Rich people decide the rules about what is masculine and what is feminine.	0.41	—	—	—
Com5	TV and newspapers want to change people’s minds about what it means to be male or female.	0.75	0.29	5.37	<0.001
Com6	International organizations are trying to create confusion about what it means to be male or female.	0.68	0.29	4.53	<0.001
Com7	Changes in the roles of men and women are being imposed by those in power.	0.70	0.22	6.19	<0.001
Com8	There is a global movement aiming to eliminate the natural distinctions between men and women.	0.57	0.28	4.27	<0.001
Values					
Val1	The family unit functions best when men and women take on distinct roles	0.74	—	—	—
Val5	I consider marriage between a man and a woman to be an important value for our society.	0.68	0.08	11.53	<0.001
Val6	For me, the distinction between male and female is a natural and unchanging reality.	0.83	0.09	13.18	<0.001
Val7	I believe that cultural and moral traditions relating to the family should be respected and passed on.	0.55	0.10	8.34	<0.001
Education					
Ed1R	Addressing gender diversity at school contributes to the development of empathy in students	0.61	—	—	—
Ed3	Discussing gender-related issues at school risks causing confusion among children.	0.35	0.14	3.66	<0.001
Ed4	I believe that gender education in schools should reflect traditional family values	0.63	0.16	6.45	<0.001
Ed6R	Teaching new perspectives on male and female roles enriches education.	0.77	0.12	9.40	<0.001
Ed8R	Schools should update their content on gender to reflect current scientific knowledge	0.62	0.14	6.23	<0.001

*N* = 263. *β* = standardized factor loading. SE and z refer to the unstandardized solution; the first indicator of each factor was fixed to 1.00 in order to set the metric of the latent variable, so that no standard error is estimated for it (—). Items ending in R are reverse-scored.

**Figure 1:**
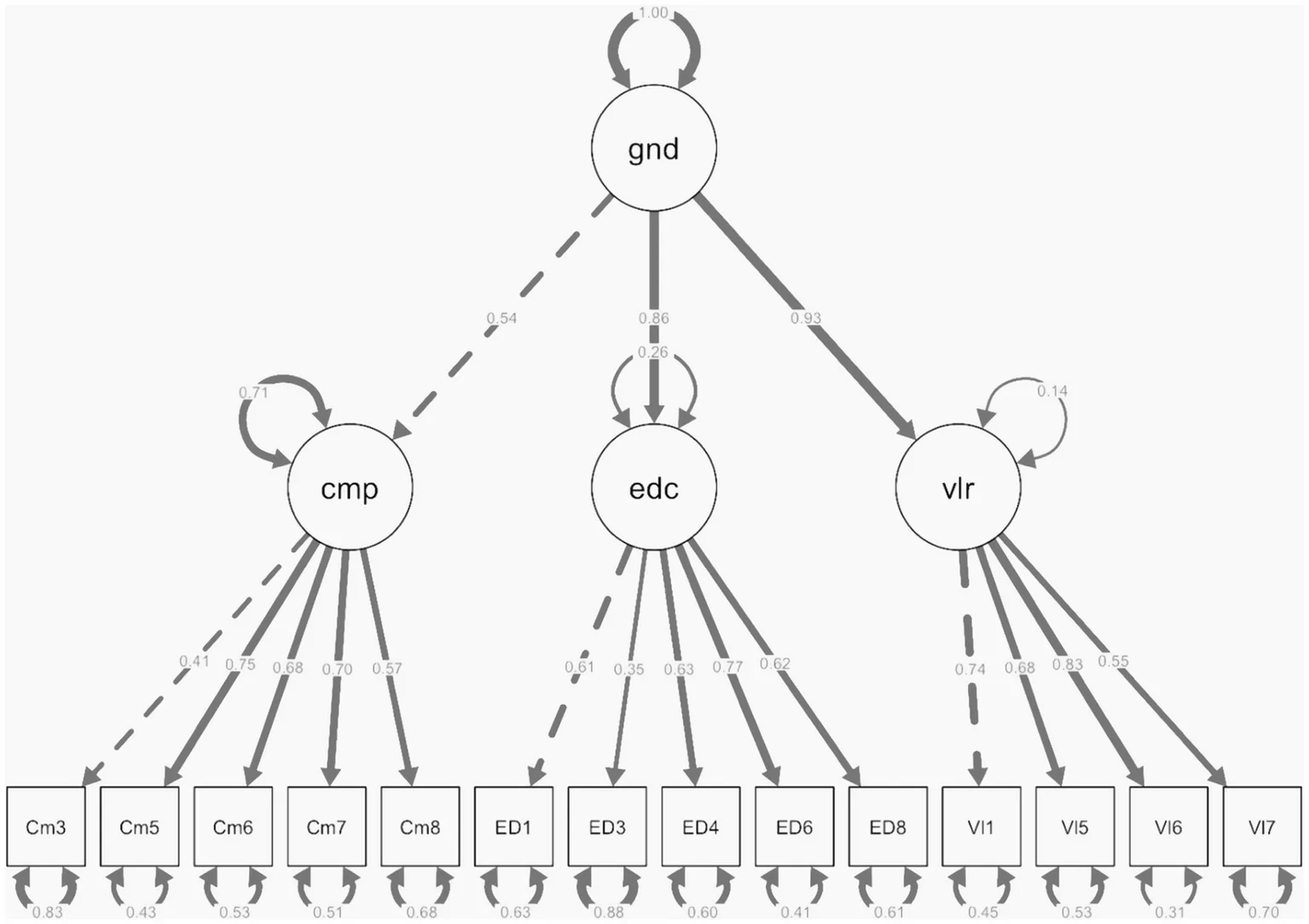
Standardized path diagram of the second-order factor model. CMP, Conspiracy factor; EDC, Education factor; VLR, Values factor; GND, Gender ideology factor.

The removal of Val2R did not substantially alter the overall structure but improved parsimony and helped address concerns about item-level performance, providing further support for the robustness of the hierarchical model. Considering these findings, item Val2R was removed from the scale and not considered in the subsequent analyses.

##### Justification of the hierarchical structure

The GIBS was designed from the outset as a three-dimensional instrument. The item pool was written to represent three domains of anti-gender discourse, and the construct definition treats those domains as facets of a single ideological orientation rather than as three independent attitudes. The measurement model tested here is therefore a translation of that definition, and not a specification selected among competitors on the basis of fit. One implication of this position deserves to be made explicit.

With exactly three first-order factors, the second-order model imposes no additional restriction on the covariance structure and is statistically equivalent to a model in which the three factors are simply allowed to correlate; the two specifications necessarily yield the same fit (Chen et al., 2006; Rindskopf and Rose, 1988). The choice between them is therefore theoretical rather than statistical, and we adopt the second-order specification for three reasons: it is the direct expression of our construct definition, in which the three domains are facets of a superordinate anti-gender orientation (Johnson et al., 2011); it makes the strength of each domain’s relation to the general construct an estimable quantity, so that the finding that conspiracy is more loosely tied to the general factor (λ = 0.54) than values (λ = 0.93) or education (λ = 0.86) is itself a substantive result rather than an artefact of parameterization; and it licenses the interpretation of the total GIBS score as an estimate of a common latent factor, which is what applied users of the scale will require.

As regards the absolute level of fit, the values obtained for the retained model (CFI = 0.903, TLI = 0.880, RMSEA = 0.076, SRMR = 0.064) fall slightly below the cutoffs commonly cited after Hu and Bentler (1999). Those cutoffs, however, were derived under specific simulation conditions and were never intended as universal thresholds; incremental indices such as CFI and TLI are known to be penalized in models with a larger number of indicators and loadings of moderate magnitude, and their mechanical application has been repeatedly cautioned against (Kenny and McCoach, 2003; Lai and Green, 2016; Marsh et al., 2004). In the present case all loadings were statistically significant and, with the single exception discussed below, of adequate magnitude; the upper bound of the RMSEA confidence interval remained below 0.10, the value conventionally taken to indicate poor fit (Browne and Cudeck, 1993); and the theoretically specified structure was reproduced without recourse to *post hoc* modifications, no correlated residuals or cross-loadings having been added at any stage. We therefore regard the hierarchical solution as providing an adequate account of the data for a newly developed multidimensional measure.

A final consideration concerns item-level performance. Besides Val2R, which was removed for the reasons detailed above, item Ed3 (“Discussing gender-related issues at school risks causing confusion among children”) showed the lowest standardized loading in the retained model (0.35). We chose to retain it on content-validity grounds, since it expresses the child-protection claim that is arguably the single most recurrent argument in public anti-gender discourse, and since its removal would have left the education factor without a directly worded indicator of that claim, the remaining items of that factor being largely reverse-worded. Its loading, although modest, is statistically significant and exceeds the threshold adopted at the item-selection stage.

Reliability analyses were conducted separately for each first-order factor. Cronbach’s α coefficients were 0.76 for conspiracy factor, 0.79 for values factor, and 0.72 for education factor, indicating acceptable internal consistency. McDonald’s Ω values showed improved values, with 0.84 for conspiracy factor, 0.81 for values factor, and 0.79 for education factor. Regarding the second-order factor, we computed the composite reliability (CR), based on the standardized loadings and residual variances of the three first-order factors. The resulting value (CR = 0.83) indicated good internal consistency at the higher-order level as well.

#### Construct validation of the GIBS scale

Correlation analyses provided evidence for the construct validity of the GIBS scale, suggesting that it captures a specific ideological construct while maintaining significant relationships with theoretically relevant variables. As reported in Table 3, GIBS was correlated with right-wing authoritarianism (*r* = 0.68, *p* < 0.001), modern prejudice (*r* = 0.75, *p* < 0.001), and overt prejudice (*r* = 0.63, *p* < 0.001). In addition, GIBS demonstrated a positive correlation with general conspiracist beliefs (*r* = 0.30, *p* < 0.001), supporting its hypothesized link with conspiracy thinking while distinguishing it from broader conspiracy content. These findings collectively support the construct validity of the GIBS scale, confirming that it reliably measures a distinct ideological dimension closely related to prejudice and authoritarian attitudes, yet sufficiently unique to be differentiated from general conspiracist beliefs. This evidence underscores the scale’s utility for capturing specific ideological nuances relevant to the studied phenomena.

**Table 3:** Correlations among the study variables (Study 2).

Variable	GIBS	RWA	MHS-G	ATG-S	GCBS	PrejTot
GIBS	1					
RWA	0.68^***^	1				
MHS-G	0.75^***^	0.73^***^	1			
ATG-S	0.63^***^	0.58^***^	0.66^***^	1		
GCBS	0.30^***^	0.37^***^	0.27^***^	0.18^***^	1	
PrejTot	0.77^***^	0.74^***^	0.98^***^	0.81^***^	0.26^***^	1

*N* = 263. ***p < 0.001. GIBS, Gender Ideology Belief Scale; RWA, Right-Wing Authoritarianism; MHS-G, Modern Homonegativity Scale – Gay men; ATG-S, Attitudes Toward Gay Men Scale (old-fashioned homonegativity); GCBS, Generic Conspiracist Beliefs Scale; PrejTot, total sexual prejudice, the composite of MHS-G and ATG-S. The magnitude of the correlation between PrejTot and MHS-G reflects the fact that the MHS-G contributes 12 of the 17 items of the composite.

#### Criterion validity

To evaluate the criterion validity of the GIBS scale, a stepwise forward linear regression was conducted using a set of relevant demographic and psychological predictors. The dependent variable was the total GIBS score, and the pool of candidate predictors included gender, age, level of education, sexual orientation, religiosity, political orientation, right-wing authoritarianism (RWA), general conspiracist beliefs (GCBS), and total sexual prejudice.

The analysis began with a null model including only the intercept and proceeded by iteratively adding variables that resulted in the greatest reduction in the Akaike Information Criterion (AIC). The final model retained five predictors: sexual prejudice, RWA, gender, GCBS, and religiosity. This model accounted for a substantial proportion of variance in GIBS scores, R^2^ = 0.63, *F*(5, 256) = 89.05, *p* < 0.001.

The standardized regression coefficients indicated that total prejudice was the most robust predictor of GIBS scores (*β* = 0.57, *p* < 0.001), followed by RWA (*β* = 0.23, *p* < 0.001). Gender was also a significant predictor, with male participants reporting higher levels of GIBS (*β* = 0.08, *p* = 0.043). GCBS showed a modest yet significant association (*β* = 0.08, *p* = 0.049). Religiosity, entered as a dummy variable comparing Christian affiliation to other categories, was not a significant predictor in the final model (*β* = −0.06, *p* = 0.125), although it contributed to improved model fit during the selection process.

The exclusion of political orientation, education, age, and sexual orientation from the final model suggests that GIBS captures ideological variance that is more strongly associated with prejudice, authoritarianism, and conspiracy beliefs than with sociodemographic characteristics. These results provide additional support for the criterion validity of the GIBS scale, showing that it systematically covaries with established constructs in the domain of ideological belief systems, while retaining specificity and explanatory power (Table 4).

**Table 4:** Stepwise forward linear regression predicting the GIBS total score (Study 2).

Variable	*β*	SE	*t*	*p*
PrejTot	0.57	0.04	9.54	<0.001
RWA	0.23	0.05	3.86	<0.001
SexM	0.08	0.87	2.03	0.043
GCBS	0.08	0.02	1.98	0.049
ChriRelig	−0.06	0.77	−1.54	0.125

*N* = 262 (one participant had missing data on at least one predictor). *β* = standardized regression coefficient; SE refers to the unstandardized coefficient. R^2^ = 0.63, *F*(5, 256) = 89.05, *p* < 0.001. SexM = male gender (reference category: female); ChriRelig = Christian affiliation (reference category: all other categories). Predictors are listed in their order of entry into the model.

To evaluate the stability and generalizability of the linear regression model predicting GIBS score from five predictors, a bootstrap cross-validation procedure was performed. This approach involves repeatedly resampling the original dataset with replacement, fitting the model on each resample, and assessing its predictive performance on the out-of-bag observations not included in the bootstrap sample (Tibshirani and Efron, 1993). This method provides a robust estimate of model performance and accounts for sampling variability.

The bootstrap procedure was implemented using 5,000 resamples via the caret package in R (Kuhn, 2008). Performance metrics averaged across all bootstrap samples included the Root Mean Square Error (RMSE), Mean Absolute Error (MAE), and coefficient of determination (R^2^). The results indicated a mean RMSE of 5.98, suggesting that on average the model’s predicted values deviated from the observed GIBS scores by approximately 6 units. The mean MAE of 4.72 further confirmed the average absolute prediction error magnitude. Importantly, the mean R^2^ value of 0.61 reflected that approximately 61% of the variance in GIBS score was explained by the predictors across resamples, indicating good explanatory power.

These bootstrap estimates suggest that the regression model maintains stable predictive performance and is not overly sensitive to specific sampling variations within the dataset. The relatively narrow error metrics and consistent R^2^ support the model’s reliability and its potential generalizability to similar samples beyond the original dataset. Bootstrap cross-validation thus complements traditional model evaluation techniques by providing an internal validation mechanism that accounts for overfitting and sampling noise (Harrell, 2015).

Overall, the bootstrap cross-validation procedure confirmed the model’s robustness and predictive validity, reinforcing confidence in the identified predictors as meaningful contributors to the ideological construct measured by GIBS.

## General discussion

The present research introduced and validated the Gender Ideology Belief Scale (GIBS), a multidimensional instrument designed to measure opposition to gender-related social change. Across two studies involving Italian participants (*N* = 595), the scale demonstrated a robust three-factor second-order structure capturing conspiratorial framing, resistance to gender-inclusive education, and adherence to traditional family values, three themes that consistently emerge in contemporary anti-gender discourse across European political contexts.

The psychometric properties of the GIBS were largely satisfactory. Reliability estimates were acceptable to good across both studies, with McDonald’s Ω values consistently outperforming Cronbach’s α, confirming the scale’s internal coherence. The second-order structure proved empirically defensible, with the higher-order gender ideology factor showing particularly strong associations with the Values (λ = 0.93) and Education (λ = 0.86) subscales, and a moderate, though meaningful, association with the Conspiracy subscale (λ = 0.54). This pattern suggests that while conspiratorial thinking is part of the anti-gender ideological constellation, it retains a degree of specificity that distinguishes it from the more normatively grounded dimensions of the construct.

Evidence for construct validity was compelling. The GIBS correlated strongly with right-wing authoritarianism (*r* = 0.68), modern sexual prejudice (*r* = 0.75), and overt homonegativity (*r* = 0.63), while maintaining a more moderate association with generic conspiracist beliefs (*r* = 0.30). This pattern of convergent and discriminant relationships confirms that the GIBS captures a specific ideological construct, one that is related to, yet distinguishable from, broader authoritarian and conspiracist orientations. Crucially, the strong overlap with sexual prejudice underscores that opposition to gender ideology is not merely a matter of abstract belief, but is deeply entangled with negative attitudes toward LGBTQIA+ individuals.

These findings align with theoretical accounts that frame anti-gender movements not as isolated moral reactions, but as expressions of a coherent ideological worldview organized around threats to traditional hierarchies, national identity, and family structures. By operationalizing this worldview as a measurable psychological construct, the GIBS offers a novel empirical tool for researchers investigating cultural backlash, norm contestation, and resistance to progressive social change. Importantly, the scale moves beyond reductionist or pathologizing approaches, treating anti-gender beliefs as a structured meaning-making system that warrants systematic psychological investigation.

Situating the instrument within the existing quantitative literature clarifies what it adds. Research based on cross-national surveys has established that gender has become a salient axis of value conflict and, in several countries, of polarization (Norris and Inglehart, 2019; Peral et al., 2024; Ural, 2026), but it has necessarily relied on legacy indicators of gender-role traditionalism that predate contemporary anti-gender mobilization and that treat the domain as unidimensional. The GIBS is not an alternative to those indicators. It is designed to capture what they cannot, namely the specific claims through which opposition to gender-related change is currently articulated, and it can therefore be used alongside them to test whether backlash is driven by generalized traditionalism, by the belief system measured here, or by their interaction. Because the three subscales can be examined separately, the scale also makes it possible to ask which component of anti-gender belief carries the association with a given outcome, a question that single-indicator measures cannot address.

### Limitations and future directions

Several limitations of the present research should be acknowledged, as they also point to important avenues for future inquiry. First, both studies relied on snowball sampling conducted through online networks, which introduces a systematic selection bias whose consequences deserve to be spelled out. Recruitment through the personal networks of an initial pool of participants tends to reproduce the social and ideological composition of those networks, so that respondents holding stronger anti-gender beliefs, who are on average older, less digitally connected, and less highly educated, are likely to be underrepresented in both samples. The descriptive statistics are consistent with this reading: item means ranged from 1.57 to 3.00 on a five-point scale, indicating that the upper end of the GIBS distribution was sparsely populated. Three distinct consequences follow. First, restriction of range at the upper end of the distribution attenuates rather than inflates the observed associations, which implies that the correlations reported here with right-wing authoritarianism, sexual prejudice, and conspiracist beliefs should be read as conservative estimates of the corresponding population values. Second, and in the opposite direction, ideologically homogeneous samples can yield factor solutions that are cleaner than those obtainable in the general population, because the covariation among items is generated by a comparatively narrow segment of the ideological spectrum; replication of the three-factor structure in a sample with adequate representation of high scorers is therefore a priority rather than a formality. Third, the criterion validity estimates, and in particular the proportion of variance accounted for by the regression model, are conditional on this sample composition and should not be assumed to generalize to probability-based samples. Future studies should prioritize probability-based or quota sampling and should consider deliberate oversampling of politically and religiously conservative respondents, so as to cover the full range of the construct.

Second, the gender composition of the samples, particularly in Study 2, where 69.2% of participants identified as female, may have systematically influenced the factor structure and criterion validity estimates. Given that male participants scored higher on the GIBS, the overrepresentation of female participants could have attenuated variability on the Values subscale. Replication with more gender-balanced samples is therefore encouraged before drawing firm conclusions about the scale’s psychometric invariance across gender groups. We did not attempt to test measurement invariance across gender in the present data. With 263 participants, of whom 28.1% identified as male and 2.7% as other, the male subgroup comprises fewer than 75 respondents, well below what is required to estimate a 14-indicator second-order model separately in each group with acceptable precision, and the third category is too small to be included in any group-based comparison. An invariance test conducted on these data would have been underpowered rather than informative, and we prefer to state this explicitly rather than to report a test incapable of detecting non-invariance had it been present. The practical implication is that the GIBS should not, for the moment, be used for latent-mean comparisons between women and men, and that scores obtained in samples with markedly different gender compositions should be compared with caution.

Third, the GIBS was developed and validated exclusively with Italian participants, and the item generation process was grounded in narratives prevalent in Italian and, more broadly, Southern European political discourse. Although anti-gender beliefs are widespread, the themes through which they are articulated vary considerably across cultural and institutional contexts. The GIBS is therefore not proposed as a cross-cultural instrument, and we would discourage its use outside Italy in its present form. A subsequent validation in other countries would nevertheless be a valuable development, and we would regard the following as the necessary steps: translation and back-translation with expert review of the cultural adequacy of each item; cognitive interviewing to verify that the claims referenced by the items are recognizable to respondents in the target context; and formal testing of configural, metric, and scalar invariance across countries, with alignment optimization as the preferable strategy when several groups are compared (Asparouhov and Muthén, 2014; Cheung and Rensvold, 2002; Putnick and Bornstein, 2016). Only if at least metric invariance holds can associations with other constructs be meaningfully compared across contexts, and only under scalar invariance can mean levels of anti-gender belief be compared. Partial non-invariance would itself be an informative outcome rather than a failure, since the content of anti-gender discourse varies with the religious and institutional configuration of each national context.

Fourth, the present research did not include a measure of social desirability, a notable limitation given the socially sensitive nature of the construct. The likely direction of the resulting bias is worth specifying. Because the normative climate in which the data were collected treats explicit opposition to gender-related rights as socially undesirable, underreporting is expected to be concentrated among high scorers and thus to compound the range restriction discussed above, further attenuating the observed associations. Two features of the present design partly mitigate the concern: administration was fully anonymous and computer-based, a condition consistently associated with reduced socially desirable responding in sensitive domains (Krumpal, 2013), and part of the item pool was deliberately written in the normalized, morally respectable register through which anti-gender claims circulate in public debate, which lowers the reputational cost of endorsement. These are mitigations rather than solutions, and the administration of a validated measure such as the Balanced Inventory of Desirable Responding (Paulhus, 1991) alongside the GIBS would allow the magnitude of the bias to be estimated and its uniformity across the three subscales to be tested; we would expect the values subscale, whose content lies closest to legitimate moral discourse, to be less affected than the conspiracy subscale.

Fifth, the fit of the retained model, while adequate, was not excellent (CFI = 0.903, TLI = 0.880, RMSEA = 0.076). As argued in Study 2, the hierarchical structure is a theoretical commitment rather than a specification chosen on the basis of fit, and we did not look for a better-fitting but theoretically inappropriate alternative. We would also note, in relation to the possibility of adding further indicators, that data collection for both studies was already complete when these analyses were carried out, so that no additional item could be administered within the present research. The psychometric evidence reported here should accordingly be read as initial evidence on a newly developed instrument.

Sixth, we acknowledge that the wording of some items could be improved. Com3 (“Rich people decide the rules about what is masculine and what is feminine”) refers to economic elites, whereas the conspiratorial frame it is meant to capture concerns transnational institutional actors, and this is plausibly reflected in its comparatively modest loading (0.41); Ed3 combines a claim about the effects of classroom discussion with an evaluative term, confusion, whose meaning may vary across respondents; and Com5 refers to a conception of the media landscape that predates social media. These formulations are the ones on which the present data were collected and on which the psychometric properties reported here were estimated, and they are therefore retained as administered; refinements of this kind are best considered in the context of the cultural adaptations discussed above, where item wording is in any case revisited.

Finally, the present studies focused exclusively on attitudinal and ideological predictors, leaving unexplored the scale’s associations with behavioral outcomes. Future research would benefit from examining how GIBS scores relate to voting behavior, political activism, policy support, and willingness to engage with gender-diverse individuals, thereby strengthening the predictive validity of the scale and contributing to a more comprehensive understanding of how ideological opposition to gender change translates into concrete social and political action. We also encourage further critical reflection on the ethical and political implications of measuring belief systems that are so socially consequential and contested.

## Conclusion

The present research provides initial support for the Gender Ideology Belief Scale (GIBS) as a theoretically grounded and psychometrically promising instrument for assessing opposition to gender-related social change.

Across two studies, the findings supported a multidimensional structure centered on conspiratorial beliefs, resistance to gender-inclusive education, and adherence to traditional family values. The scale also showed meaningful associations with right-wing authoritarianism, sexual prejudice, and generic conspiracist beliefs, suggesting that anti-gender beliefs are embedded within a broader ideological network while retaining content-specific features. Although further validation is needed across more diverse and culturally heterogeneous samples, the GIBS may offer a useful tool for investigating how resistance to gender-related social change is psychologically organized and socially expressed. In this sense, the scale may contribute to future research on cultural backlash, ideological polarization, and the attitudinal foundations of contemporary conflicts over gender and diversity.

## Data Availability

The original contributions presented in the study are included in the article/Supplementary material, further inquiries can be directed to the corresponding author.
