# Supplementary material for: Unmasking the anti-gender mindset: development and validation of the Gender Ideology Belief Scale
Source: Front Psychol. 2026 Sep 16;17:1931745. doi: 10.3389/fpsyg.2026.1931745 (PMC13624096; doi:10.3389/fpsyg.2026.1931745)
Supplement: Supplementary file 1 [file Supplementary_file_1.docx]

Appendix

Table A1. *Sample’s Descriptive (Study 1)*

| *Variable* | *N* | *f* | *M* | *SD* |
| --- | --- | --- | --- | --- |
| **Gender** |  |  |  |  |
| Male | 142 | 42.4% |  |  |
| Female | 188 | 56.1% |  |  |
| Other | 5 | 1.5% |  |  |
| **Age** |  |  | 31.48 | 12.18 |
| **Sexual Orientation** |  |  |  |  |
| Exclusively Heterosexual | 281 | 83.6% |  |  |
| Bisexual | 23 | 6.8% |  |  |
| Exclusively Homosexual | 17 | 5.1% |  |  |
| Do not answer | 15 | 4.5% |  |  |
| **Educational Level** |  |  |  |  |
| Elementary School Diploma | 2 | 0.6% |  |  |
| Middle School Diploma | 6 | 1.8% |  |  |
| High School Diploma | 134 | 40.2% |  |  |
| Bachelor’s Degree | 104 | 31.2% |  |  |
| Master’s Degree | 62 | 18.6% |  |  |
| Phd or Other Post-Degree | 25 | 7.5% |  |  |
| **Political Orientation** |  |  | 3.90 | 1.93 |

Note. Political orientation was measured with a single item on a 9-point scale ranging from 1 = extreme left to 9 = extreme right.

Table A2. Descriptive of the original pool of 24 items of GIBS *(Study 1)*

| *Item* | *Item content* | *M* | *SD* | *Skew* | *Kurtosis* |
| --- | --- | --- | --- | --- | --- |
| Com1R | The fight for the rights of gender-diverse people is not controlled by secret forces or hidden governments. | 2.02 | 1.27 | 1.05 | -0.01 |
| Com2 | Some international organizations want to create confusion about what it means to be a man or a woman. | 2.18 | 1.29 | 0.79 | -0.48 |
| Com3 | Rich people decide the rules about what is masculine and what is feminine. | 2.24 | 1.33 | 0.69 | -0.75 |
| Com4 | Media that talk about changing how we see gender want to manipulate society and make it weaker. | 1.96 | 1.26 | 1.19 | 0.37 |
| Com5 | TV and newspapers want to change people’s minds about what it means to be male or female. | 1.63 | 1.11 | 1.84 | 2.49 |
| Com6 | International organizations are trying to create confusion about what it means to be male or female. | 1.96 | 1.24 | 1.10 | 0.12 |
| Com7 | Changes in the roles of men and women are being imposed by those in power. | 1.92 | 1.24 | 1.19 | 0.28 |
| Com8 | There is a global movement aiming to eliminate the natural distinctions between men and women. | 1.58 | 1.04 | 1.85 | 2.69 |
| Val1 | The family unit functions best when men and women take on distinct roles | 1.99 | 1.33 | 1.09 | -0.18 |
| Val2R | Believing that gender identities other than male and female exist can be compatible with religious traditions. | 2.81 | 1.17 | 0.01 | -0.66 |
| Val3R | Talking about different genders helps to enhance traditional roles of men and women. | 3.17 | 1.30 | -0.23 | -0.95 |
| Val4 | Changing gender roles risks dividing the community. | 1.85 | 1.21 | 1.27 | 0.45 |
| Val5 | I consider marriage between a man and a woman to be an important value for our society. | 1.94 | 1.34 | 1.15 | -0.06 |
| Val6 | For me, the distinction between male and female is a natural and unchanging reality. | 1.94 | 1.38 | 1.22 | 0.03 |
| Val7 | I believe that cultural and moral traditions relating to the family should be respected and passed on. | 1.85 | 1.28 | 1.28 | 0.34 |
| Val8R | Accepting diverse gender identities helps protect the traditional family. | 2.16 | 1.30 | 0.81 | -0.57 |
| Ed1R | Addressing gender diversity at school contributes to the development of empathy in students | 1.68 | 1.19 | 1.72 | 1.76 |
| Ed2R | Inclusive education in schools helps children understand and respect the differences between boys and girls. | 2.09 | 1.32 | 0.97 | -0.28 |
| Ed3 | Discussing gender-related issues at school risks causing confusion among children. | 1.74 | 1.20 | 1.50 | 1.04 |
| Ed4 | I believe that gender education in schools should reflect traditional family values | 1.94 | 1.19 | 1.07 | 0.10 |
| Ed5 | Children with a different gender identity should be invisible at school. | 1.40 | 1.04 | 2.58 | 5.38 |
| Ed6R | Teaching new perspectives on male and female roles enriches education. | 1.87 | 1.25 | 1.33 | 0.61 |
| Ed7 | Telling children that gender can be chosen confuses them and makes them feel bad. | 2.06 | 1.43 | 1.00 | -0.47 |
| Ed8R | Schools should update their content on gender to reflect current scientific knowledge. | 2.38 | 1.27 | 0.61 | -0.61 |

Fig.A1 Scree plot of the 23 items of GIBS.

Table A3. Loadings (*λ*) and Communalities (*h^2^*) of the 23 items of GIBS included in the EFA cut to .20.

| *Item* | *Item content* | *MR1* | *MR2* | *MR3* | *h^2^* |
| --- | --- | --- | --- | --- | --- |
| Com1R | The fight for the rights of gender-diverse people is not controlled by secret forces or hidden governments. | 0.42 |  | 0.21 | 0.42 |
| Com2 | Some international organizations want to create confusion about what it means to be a man or a woman. | 0.66 |  |  | 0.47 |
| Com3 | Rich people decide the rules about what is masculine and what is feminine. | 0.72 |  |  | 0.34 |
| Com4 | Media that talk about changing how we see gender want to manipulate society and make it weaker. | 0.66 |  |  | 0.56 |
| Com5 | TV and newspapers want to change people’s minds about what it means to be male or female. | 0.89 |  |  | 0.74 |
| Com6 | International organizations are trying to create confusion about what it means to be male or female. | 0.71 |  |  | 0.38 |
| Com7 | Changes in the roles of men and women are being imposed by those in power. | 0.75 |  |  | 0.59 |
| Com8 | There is a global movement aiming to eliminate the natural distinctions between men and women. | 0.75 |  |  | 0.77 |
| Val1 | The family unit functions best when men and women take on distinct roles |  | 0.59 | 0.24 | 0.67 |
| Val2R | Believing that gender identities other than male and female exist can be compatible with religious traditions. |  | 0.73 |  | 0.39 |
| Val3R | Talking about different genders helps to enhance traditional roles of men and women. |  | 0.55 | -0.20 | 0.55 |
| Val4 | Changing gender roles risks dividing the community. | 0.21 | 0.57 |  | 0.64 |
| Val5 | I consider marriage between a man and a woman to be an important value for our society. |  | 0.61 |  | 0.69 |
| Val6 | For me, the distinction between male and female is a natural and unchanging reality. | 0.21 | 0.65 |  | 0.73 |
| Val7 | I believe that cultural and moral traditions relating to the family should be respected and passed on. | 0.21 | 0.63 |  | 0.66 |
| Val8R | Accepting diverse gender identities helps protect the traditional family. |  | 0.54 | 0.31 | 0.49 |
| Ed1R | Addressing gender diversity at school contributes to the development of empathy in students |  |  | 0.81 | 0.67 |
| Ed2R | Inclusive education in schools helps children understand and respect the differences between boys and girls. |  |  | 0.73 | 0.64 |
| Ed3 | Discussing gender-related issues at school risks causing confusion among children. |  |  | 0.93 | 0.69 |
| Ed4 | I believe that gender education in schools should reflect traditional family values | -0.22 |  | 0.90 | 0.60 |
| Ed6R | Teaching new perspectives on male and female roles enriches education. |  |  | 0.78 | 0.64 |
| Ed7 | Telling children that gender can be chosen confuses them and makes them feel bad. |  |  | 0.72 | 0.69 |
| Ed8R | Schools should update their content on gender to reflect current scientific knowledge |  |  | 0.76 | 0.49 |

Fig.A2 Scree plot of the 15 items of GIBS.

Fig. A3. Power Analysis for CFA Model

Table A4. *Sample’s Descriptive (Study 2)*

| *Variable* | *N* | *f* | *M* | *SD* |
| --- | --- | --- | --- | --- |
| **Gender** |  |  |  |  |
| Male | 74 | 28.1% |  |  |
| Female | 182 | 69.2% |  |  |
| Other | 7 | 2.7% |  |  |
| **Age** |  |  | 30.61 | 13.48 |
| **Sexual Orientation** |  |  |  |  |
| Exclusively Heterosexual | 206 | 78.3% |  |  |
| Bisexual | 28 | 10.6% |  |  |
| Exclusively Homosexual | 29 | 11.0% |  |  |
| **Educational Level** |  |  |  |  |
| Middle School Diploma | 18 | 6.8% |  |  |
| High School Diploma | 134 | 51.0% |  |  |
| Bachelor’s Degree | 34 | 12.9% |  |  |
| Master’s Degree | 70 | 26.6% |  |  |
| PhD or Other Post-Degree | 7 | 2.7% |  |  |
| **Political Orientation** |  |  | 3.62 | 1.55 |

Table A5. Descriptive of 15 items of GIBS *(Study 2)*

| *Item* | *M* | *SD* | *Skew* | *Kurtosis* |
| --- | --- | --- | --- | --- |
| Ed1R | 1.92 | 1.20 | 1.17 | 0.36 |
| Ed3 | 2.15 | 1.08 | 0.74 | -0.18 |
| Ed4 | 1.87 | 1.22 | 1.36 | 0.77 |
| Ed6R | 1.66 | 1.07 | 1.75 | 2.32 |
| Ed8R | 1.57 | 1.01 | 1.95 | 3.23 |
| Val1 | 1.73 | 1.19 | 1.61 | 1.45 |
| Val2R | 3.00 | 1.30 | 0.00 | -0.94 |
| Val5 | 1.78 | 1.24 | 1.48 | 0.91 |
| Val6 | 1.82 | 1.23 | 1.38 | 0.77 |
| Val7 | 2.01 | 1.31 | 0.99 | -0.36 |
| Com3 | 2.40 | 1.44 | 0.47 | -1.20 |
| Com5 | 2.08 | 1.22 | 0.78 | -0.51 |
| Com6 | 1.94 | 1.15 | 1.09 | 0.34 |
| Com7 | 1.92 | 1.17 | 1.07 | 0.12 |
| Com8 | 2.03 | 1.25 | 0.95 | -0.16 |

Table A6. Final fourteen-item version of the GIBS

| *Item/factor* | *Item content English* | *Item content Italian* |
| --- | --- | --- |
| **Conspiracy** |  |  |
| Com3 | Rich people decide the rules about what is masculine and what is feminine. | Le persone ricche decidono le regole su cosa è maschile e cosa è femminile |
| Com5 | TV and newspapers want to change people’s minds about what it means to be male or female. | La TV e i giornali vogliono far cambiare idea alle persone su cosa significa essere maschio o femmina. |
| Com6 | International organizations are trying to create confusion about what it means to be male or female. | Le organizzazioni internazionali stanno cercando di creare confusione su cosa significhi essere maschio o femmina. |
| Com7 | Changes in the roles of men and women are being imposed by those in power. | I cambiamenti nei ruoli tra maschi e femmine vengono imposti da chi comanda |
| Com8 | There is a global movement aiming to eliminate the natural distinctions between men and women. | Esiste un movimento globale con l’obiettivo di eliminare le distinzioni naturali tra uomini e donne. |
| **Values** |  |  |
| Val1 | The family unit functions best when men and women take on distinct roles. | Il nucleo familiare funziona meglio quando uomini e donne ricoprono ruoli distinti |
| Val5 | I consider marriage between a man and a woman to be an important value for our society. | Considero il matrimonio tra uomo e donna un valore importante per la nostra società. |
| Val6 | For me, the distinction between male and female is a natural and unchanging reality. | Per me, la distinzione tra maschile e femminile è una realtà naturale e immutabile. |
| Val7 | I believe that cultural and moral traditions relating to the family should be respected and passed on. | Ritengo che le tradizioni culturali e morali sulla famiglia vadano rispettate e tramandate. |
| **Education** |  |  |
| Ed1R | Addressing gender diversity at school contributes to the development of empathy in students | Affrontare la diversità di genere a scuola contribuisce allo sviluppo dell'empatia negli studenti. |
| Ed3 | Discussing gender-related issues at school risks causing confusion among children. | Trattare temi legati al genere a scuola rischia di creare confusione nei bambini. |
| Ed4 | I believe that gender education in schools should reflect traditional family values | Credo che l'educazione scolastica sul genere debba rispecchiare i valori tradizionali della famiglia |
| Ed6R | Teaching new perspectives on male and female roles enriches education. | Insegnare nuove prospettive sui ruoli maschili e femminili arricchisce l’educazione. |
| Ed8R | Schools should update their content on gender to reflect current scientific knowledge | La scuola dovrebbe adeguare i propri contenuti sul genere alle conoscenze scientifiche attuali. |

**Supplementary Material S1. Item generation procedure**

The items for the Gender Ideology Belief Scale (GIBS) were developed through a multi-phase procedure aimed at maximizing theoretical coherence, content relevance, and ecological validity. In the first phase, we conducted a comprehensive review of the literature on anti-gender discourse, drawing on contributions from social psychology, political psychology, gender studies, and adjacent fields concerned with ideological resistance to social change. This review made it possible to identify the main themes, symbolic oppositions, and recurrent rhetorical patterns underlying negative representations of gender ideology, as well as to situate these representations within broader belief systems related to tradition, hierarchy, and moral threat. Particular attention was devoted to theoretical and empirical work describing how opposition to gender-related social change is often articulated through concerns about the erosion of natural sex differences, the protection of children, and the preservation of the traditional family.

In the second phase, we complemented the academic review with an analysis of publicly available materials reflecting anti-gender discourse in naturalistic settings. These materials included political speeches, media reports, manifestos produced by anti-gender movements, and statements disseminated by religious and conservative advocacy groups. The aim of this phase was to identify the arguments, claims, and narrative frames most frequently mobilized in public opposition to gender-related social change, thus ensuring that the scale would not rely exclusively on abstract theorization but would also reflect the language and meanings circulating in real-world debates. This inductive examination proved particularly useful for capturing the concrete discursive expressions through which anti-gender beliefs are communicated, legitimized, and socially reproduced. The analysis of these materials further reinforced the relevance of three recurring domains, namely conspiratorial interpretations of gender ideology, resistance to gender-inclusive education, and the defence of traditional family and moral values.

On the basis of the evidence gathered in the first two phases, the third phase focused on generating an initial pool of items intended to represent the content breadth of the construct. Items were written to reflect each of the three theoretically grounded dimensions, while also capturing variability in tone and intensity, ranging from explicit conspiratorial claims to more socially acceptable or culturally normalized expressions of concern about education, morality, and family order. In drafting the items, care was taken to preserve conceptual specificity, avoid excessive redundancy, and ensure that statements remained understandable, concise, and suitable for administration in a self-report format. This stage resulted in a broad preliminary item pool designed to provide adequate coverage of the multidimensional nature of anti-gender beliefs.

In the fourth phase, the preliminary item pool was submitted to expert evaluation involving scholars with expertise in psychology, political science, and gender studies. The purpose of this review was to assess the conceptual clarity, representativeness, and theoretical consistency of each item, as well as to identify possible sources of ambiguity, overlap, or unintended bias. Experts were invited to provide qualitative feedback on the wording of the items, their correspondence with the intended dimensions, and their ability to capture ideologically meaningful content without introducing unnecessary complexity. On the basis of this feedback, several items were revised, re-worded, or discarded in order to improve precision and reduce redundancy. This iterative process led to a refined set of 24 items that were judged to be more clearly aligned with the theoretical framework and more suitable for subsequent psychometric testing.
